# Jugular Venous Catheterization is Not Associated with Increased Complications in Patients with Aneurysmal Subarachnoid Hemorrhage

**DOI:** 10.1007/s12028-024-02173-1

**Published:** 2024-11-26

**Authors:** Feras Akbik, Yuyang Shi, Steven Philips, Cederic Pimentel-Farias, Jonathan A. Grossberg, Brian M. Howard, Frank Tong, C. Michael Cawley, Owen B. Samuels, Yajun Mei, Ofer Sadan

**Affiliations:** 1 Division of Neurocritical Care, Department of Neurology and Neurosurgery, Emory University School of Medicine, 1364 Clifton Rd NE, Atlanta, GA 30322 USA; 2 H. Milton Stewart School of Industrial and Systems Engineering, Georgia Institute of Technology, Atlanta, GA USA; 3 Department of Neurosurgery, Emory University Hospital and School of Medicine, Atlanta, GA USA

**Keywords:** Subarachnoid hemorrhage, Aneurysm, Intracranial pressure, Central venous catheterization

## Abstract

**Background:**

Classic teaching in neurocritical care is to avoid jugular access for central venous catheterization (CVC) because of a presumed risk of increasing intracranial pressure (ICP). Limited data exist to test this hypothesis. Aneurysmal subarachnoid hemorrhage (aSAH) leads to diffuse cerebral edema and often requires external ventricular drains (EVDs), which provide direct ICP measurements. Here, we test whether CVC access site correlates with ICP measurements and catheter-associated complications in patients with aSAH.

**Methods:**

In a single-center retrospective cohort study, patients with aSAH admitted to Emory University Hospital between January 1, 2012, through December 31, 2020, were included. Patients were assigned by the access site of the first CVC placed. The subset of patients with an EVD were further studied. ICP measurements were analyzed using linear mixed effect models, with a binary comparison between internal-jugular (IJ) versus non-IJ access.

**Results:**

A total of 1577 patients were admitted during the study period with CVC access: subclavian (SC) (887, 56.2%), IJ (365, 23.1%), femoral (72, 4.6%), and peripheral inserted central catheter (PICC) (253, 16.0%). Traumatic pneumothorax was the most common with SC access (3.0%, *p* < 0.01). Catheter-associated infections did not differ between sites. Catheter-associated deep venous thrombosis was most common in femoral (8.3%) and PICC (3.6%) access (*p* < 0.05). A total of 1220 patients had an EVD, remained open by default, generating 351,462 ICP measurements. ICP measurements, as compared over the first 24–postinsertion hours and the next 10 days, were similar between the two groups. Subgroup analysis accounting for World Federation of Neurological Surgeons grade on presentation yielded similar results.

**Conclusions:**

Contrary to classic teaching, we find that IJ CVC placement was not associated with increased ICP in the clinical context of the largest, quantitative data set to date. Further, IJ access was the least likely to be associated with an access-site complication when compared with SC, femoral, and PICC. Together, these data support the safety, and perhaps preference, of ultrasound-guided IJ venous catheterization in neurocritically ill patients.

**Supplementary Information:**

The online version contains supplementary material available at 10.1007/s12028-024-02173-1.

## Introduction

Intracranial pressure (ICP) management is a common management challenge in the neurointensive care unit (neuro-ICU). Classic teaching has advised avoidance of internal-jugular (IJ) venous catheterization when resuscitating neuro-ICU patients to avoid jeopardizing jugular venous outflow and increasing cerebral blood volume in patients with comorbid ICP problems [1–8].

Historically, this dogma has been challenging to test systematically. Certainly, venous outflow obstruction can precipitate intracranial hypertension, as in cerebral venous sinus thrombosis. However, there are only rare reports of extracranial, jugular venous catheterization precipitating changes in ICP, most commonly when larger hemodialysis catheters have been introduced for hemodialysis [9, 10]. Notably, this has been reported more commonly in appendicular fistulous access than in jugular access in patients who are at higher risk for venous stenosis [9]. Given the rarity of this observation with jugular access, differences in catheter caliber for hemodialysis, and the high rate of occult venous stenosis in patients with renal failure, it is unclear how representative this is of the broader neuro-ICU population [10, 11].

Conversely, multiple lines of inquiry have been unable to demonstrate a relationship between jugular venous access and elevations in ICP. For instance, in 50 patients who underwent jugular venous ultrasonography before and after jugular venous catheterization, there was no change in the jugular vein diameter or significant decline in venous flow rate [12]. These data argue against any significant impedance of venous outflow after jugular central venous catheterization (CVC) placement. Consistent with these observations, two small series of heterogenous adult and pediatric neurocritical care patients directly tested this relationship in patients with external ventricular drains (EVDs) [13, 14]. There was no difference in ICP immediately before or after jugular venous catheterization. There are two limitations to these reports. First, the limited sample size; second, although suggestive, these are all limited experiences and do not comment on the often-prolonged need for CVC in neuro-ICU patients and the risk of delayed venous thrombosis causing outflow obstruction.

The site of CVC access is of particular importance in neuro-ICU patients given the known complications of other access sites. Subclavian (SC) access, at least the traditional nonultrasound-guided type, has a demonstrable increased risk of pneumothorax [15]. Peripherally inserted central catheters (PICCs) significantly reduce the risk of certain complications such as infections and pneumothorax [16, 17]. Conversely, PICCs are associated with increased rates of deep venous thrombosis (DVT) in critically ill patients, thereby causing treatment dilemmas with a need for systemic anticoagulation in patients at increased risk of intracranial hemorrhagic complications [18]. Another important parameter is the prolonged neuro-ICU–specific hospitalizations and mobilization limitations, which may increase the infection risk, especially with a femoral approach [15]. The ubiquitous adoption of ultrasound guidance for jugular access has significantly reduced CVC insertion complications, which suggests that jugular access may be a reasonable first choice in neuro-ICU patients if ICP safety concerns can be better characterized.

Aneurysmal subarachnoid hemorrhage (aSAH) provides a distinct opportunity to examine the relationship between access site, ICP, and catheter-associated complications. Unlike ischemic stroke, aSAH leads to a diffuse pattern of cerebral edema due to the widespread distribution of subarachnoid blood, sidestepping questions of lesion and catheter laterality when investigating ICP outcomes. Furthermore, these patients routinely require prolonged CVC placement, EVD, and neuro-ICU care, providing a unique opportunity to characterize the relationship between CVC access site, ICP, and catheter-associated complications in a large, single-center experience. This study hypothesized that the location of CVC access site, and specifically IJ catheterization, does not alter ICP under our current standard practices.

## Methods

The data that support the findings of this study are available from the corresponding author on reasonable request. The institutional review board at Emory University, Atlanta, GA, approved the data collection and quality assurance analysis and waived the need for patient consent (IRB00008421, 12/12/2018, “Neuroscience Outcomes”). This study was conducted in accordance with the ethical standards of the Emory institutional review board and the Helsinki Declaration of 1975.

### Patient Population

This is a retrospective cohort study of all patients aged 18 or older admitted to Emory University Hospital from January 1, 2012, through December 31, 2020, as previously described [19]. All patients with a discharge diagnosis of aSAH or aneurysmal pattern angiogram-negative SAH during this time frame were screened. To be included, patients needed have a CVC placed within the first 48 h of admission, and the subset of these patients with EVDs were further analyzed to characterize the relationship between CVC location and ICP. There was no minimum duration for either device placement.

### CVC Placement and Complication Adjudication

The medical record was systematically reviewed for the placement of central inserted central catheter or PICCs. For comparisons of baseline demographics, patients were labeled by the first CVC access site.

Central venous catheters were placed in the usual fashion, as dictated by the site of access. All IJ CVCs and PICCs were ultrasound guided. Ultrasound guidance was variably used for femoral access and was not used for subclavian access in this cohort. At our institution, CVCs are typically attempted in the jugular vein, even when seated upright, before femoral access. Femoral access is typically reserved for patients in extremis. It is our center’s practice to exchange femoral CVCs for an alternative access site once the patient is clinically stable.

A CVC-associated complication was defined as a composite incidence of traumatic pneumothorax, catheter-associated DVT (caDVT), or central line associated blood stream infection (CLABSI). Catheter-associated complications were adjudicated per catheter. Given the historical aversion to placing IJ CVCs in neurocritically ill patients, patients were bifurcated along IJ versus non-IJ categories. Within the non-IJ group, subcategories included SC, femoral, and PICC.

A traumatic pneumothorax was defined by the detection of a pneumothorax on chest radiography. This was detected by reviewing every chest x-ray report within 72 h of a CVC/PICC placement and reviewing procedure reports for thoracostomy placement. The pneumothorax was attributed to a given CVC placement attempt if it was ipsilateral to the side of the attempt and was detected within 72 h of the attempt, while excluding other potential causes.

caDVT was determined by primary review of all ultrasound and angiography reports to detect DVT. The DVT was deemed to be catheter associated if (1) it occurred in the same vein as the CVC location and side, (2) was discovered after placement of the CVC, and (3) occurred while the catheter was in place. If there was evidence of a pulmonary embolus but no caDVT, this was deemed nonattributable. Pulmonary emboli were therefore not analyzed because only caDVTs were interpreted as events. Ultrasound and angiography studies to detect thrombotic complications were clinically driven and not part of routinized surveillance. Only studies that were performed after the placement of the first CVC were analyzed. At our institution, upper extremity venous ultrasounds also assess the jugular vein.

CLABSI was institutionally determined according to the National Healthcare Safety Network criteria and prospectively maintained by Emory University Hospital [20].

### ICP Analysis

A subset of 1035 patients also had an EVD placed during their admission at our institution, before the insertion of a central venous catheter. ICP measurements were taken at least hourly throughout the duration of the EVD. In our institution, the EVD remains open to drain as the default setting. Because of the confound of multiple CVCs complicating ICP analysis and comparison, we focused our analysis on whether the IJ vein was catheterized. Patients were bifurcated into those who never had IJ access versus those who had at least had one IJ catheterization within the first 48 h of admission. Beyond that distinction, the number and combination of catheterization sites differed between patients.

Two time points were used as reference for the ICP analysis: registration and insertion time. Registration time was the time the first ICP measurement was registered (i.e., when an EVD was placed). Insertion time was considered when the first CVC was placed.

### Statistical Analysis

Descriptive statistics for continuous data are presented as medians ± interquartile ranges. Ordinal data were presented as medians with interquartile ranges. Categorical data were presented as counts and percentages. To compare between two cohorts, we report the *p* values by using the Wilcoxon rank-sum test for continuous variables, Fisher’s exact test for binary variables, and the *χ*^2^ test for multinomial variables. Post hoc analysis was done with Tukey testing for pairwise comparisons between each of the three groups.

To compare the ICP curves, we first preprocessed the data as follows: ICP measurements were aligned to the admission time or insertion time, and then measurements were extracted within certain time windows of interest; next, within each time window, we applied the linear mixed effect models to compare the two cohorts and report the *p* values of the intercepts and slopes when testing the null-hypothesis that the two curves are the same lines (further detailed in the supplementary methods).

All statistical analyses were done using R studio 3.6.2.

## Results

A total of 1228 patients were admitted with aSAH requiring CVC and EVD placement. Of these patients, 285 (23.2%) required one or more additional CVCs during the hospitalization (Table 1), with 220 (17.9%) having had a second CVC that was at an access site different from the index site (Supplementary Tables S1 and S2). A total of 2789 CVCs were placed for a total of 17,343 days. Frontline choices for CVC placement were predominantly SC and then IJ. Catheter duration was comparable to IJ, SC, and PICC, whereas femoral catheters were often exchanged within several days (Supplementary Table S2).

**Table 1:** Patient demographics and clinical characteristics by CVC access site

Demographics	Internal Jugular	Subclavian Vein	Femoral	PICC	
Age	52 (44–63)	55 (46–63)	55 (47–66)	53 (42–66)	0.726
Female	205 (67.4)	571 (70.7)	28 (70)	53 (69.7)	0.778
Race					0.002
White	106 (34.9)	337 (41.7)	12 (30)	32 (42.1)	
African American	136 (44.7)	278 (34.4)	11 (27.5)	23 (30.3)	
Asian	6 (2.0)	27 (3.3)	4 (10)	4 (5.3)	
Other	49 (16.1)	145 (17.9)	13 (32.5)	12 (15.8)	
Hispanic	7 (2.3)	21 (2.6)	0 (0)	5 (6.6)	
Hypertension	176 (57.9)	458 (56.7)	21 (52.5)	44 (57.9)	0.992
Diabetes Mellitus	49 (16.1)	90 (11.1)	6 (15)	13 (17.1)	0.094
Smoker	73 (24.0)	239 (29.6)	12 (30.0)	27 (35.5)	0.149
Coronary artery disease	25 (8.2)	47 (5.8)	3 (7.5)	8 (10.5)	0.27
Hypercholesterolemia/dyslipidemia	46 (15.1)	119 (14.7)	1 (2.5)	18 (23.7)	**0.024**
Etiology					**0.17**
Aneurysmal	242 (79.6)	699 (86.5)	37 (92.5)	63 (82.9)	
Angiogram negative	62 (20.4%)	109 (13.5%)	3 (7.5)	13 (17.1)	
WFNS grade					** < .001**
1	92 (30.3)	256 (31.7)	5 (12.5)	46 (60.5)	
2	82 (27.3)	212 (26.2)	2 (5.0)	14 (18.4)	
3	21 (6.9)	42 (5.2)	2 (5.0)	5 (6.6)	
4	70 (23.0)	189 (23.4)	6 (15.0)	6 (7.9)	
5	38 (12.5)	109 (13.5)	25 (62.5)	5 (6.6)	
Surgical treatment					** < .001**
Clip ligation	75 (24.7)	195 (24.1)	7 (17.5)	17 (22.4)	
Endovascular embolization	142 (46.7)	452 (55.9)	9 (22.5)	43 (56.6)	
None	87 (28.6)	161 (19.9)	21 (52.5)	16 (21.1)	

*WFNS* World Federation of Neurological Surgeons

Patient demographics did not significantly vary with frontline catheter placement choice (Table 1, Supplementary Table S3). There was no significant difference in CVC selection for aneurysmal versus angiogram-negative nontraumatic patterns of subarachnoid hemorrhage. Patients with higher World Federation of Neurological Surgeons (WFNS) grade on presentation were more likely to have femoral catheters placed and less likely to receive PICCs (*p* < 0.001), whereas there was no correlation of WFNS grade with IJ or SC catheterization. Choice of aneurysm intervention correlated with frontline CVC choice, with only femoral access differing from IJ, SC, and PICC (*p* < 0.001, *p* < 0.01, and *p* < 0.01, respectively, Table 1).

**Fig. 1:**
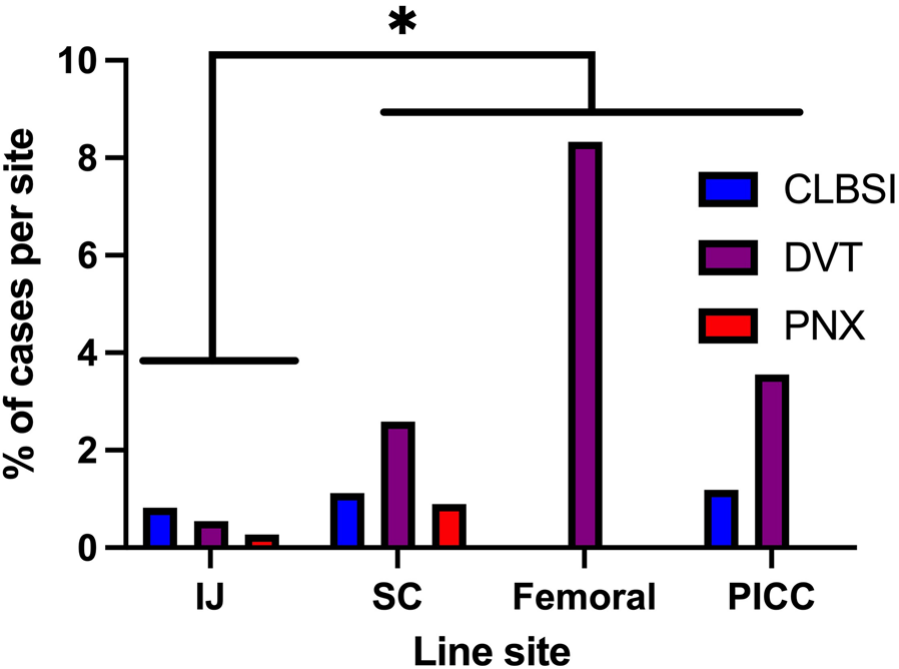
Rates of complications per central line insertion site. *p < 0.05 comparing internal jugular (IJ) vein insertion site compared to all non-IJ sites. PICC, peripherally inserted central catheter; SC, subclavian vein

Catheter-associated complications significantly varied by insertion location. IJ access was associated with significant lower total incidence of complications (1.6%) and catheter-day-adjusted-rate (0.0016 events/day) than non-IJ (4.8% and 0.0462, respectively, *p* < 0.001, Table 2, Supplementary Table S4). When analyzing specific complications per access site, SC access was associated with traumatic pneumothorax (Supplementary Table S4, Fig. 1). Furthermore, when analyzing caDVT, femoral and PICC access had higher rates (8.3% and 3.6%, respectively) compared with SC and IJ (0.9% and 0.5%, respectively, Table 2). Notably, lower extremities were assessed significantly more often than upper extremities for thrombotic complications (Supplementary Table S5). In a binary logistic regression comparing all sites to IJ, placing a femoral line increased the risk for DVT with an odds ratio (OR) of 14.8 (95% confidence interval [CI] 2.8–77.9), whereas PICC placement increased it with an odds ratio of 7.2 (95% confidence interval 1.2–43.9) (Supplementary Table S6). CLABSI rates did not differ between sites, although the total incidence was low (0.8%).

To assess whether IJ catheterization modified ICP, we analyzed the hourly ICP measurements in patients who underwent both EVD and CVC placement within the first 48 h of admission. 1035 patients with 350,412 ICP measurements were included and grouped by those who had IJ catheterization versus that never had IJ catheterization (non-IJ, Supplementary Table S3).

**Fig. 2:**
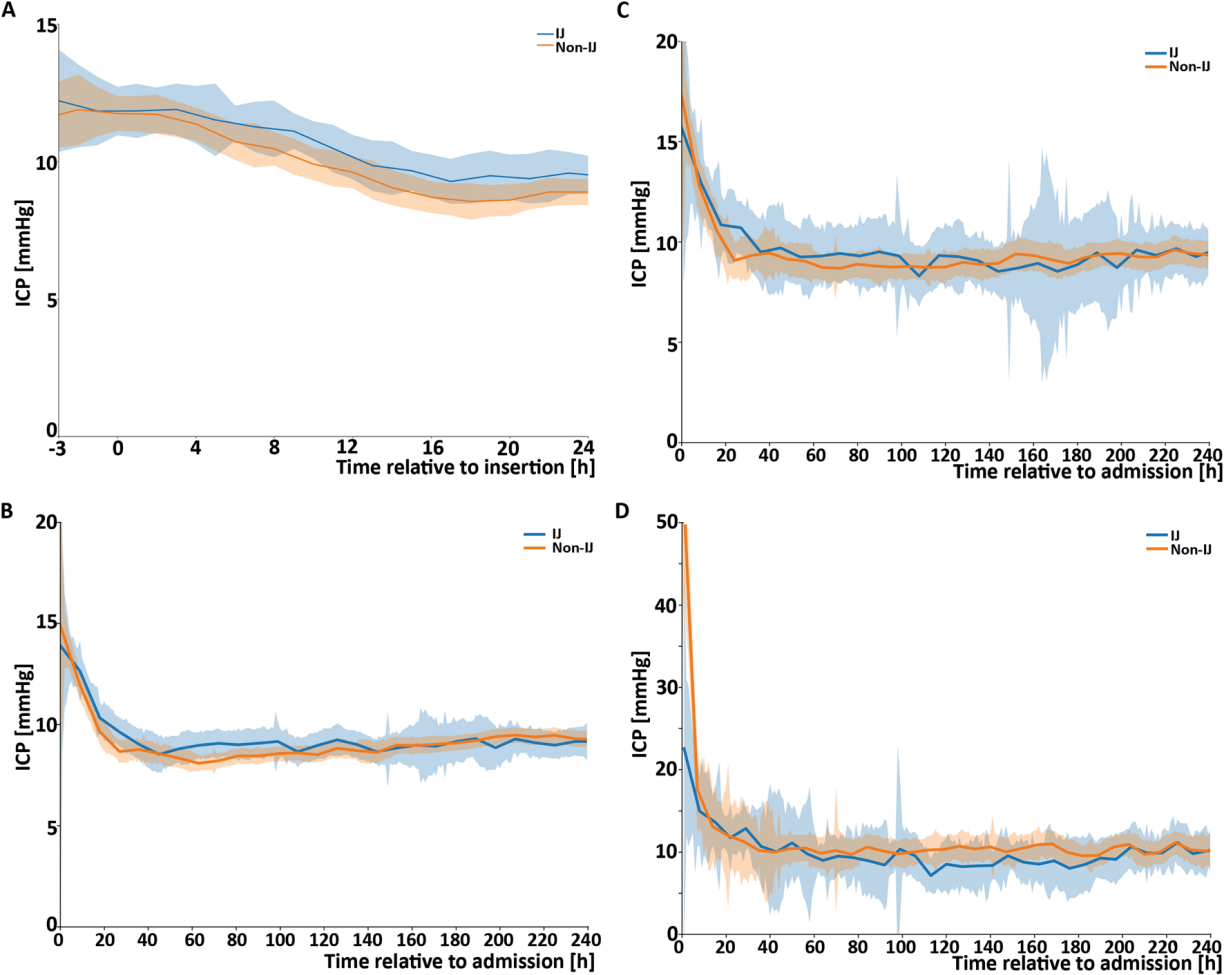
Changes in absolute ICPs in a patient who had an internal jugular (IJ) site central line insertion versus all other sites (non-IJ: subclavian, femoral veins, and peripherally inserted central line together). **a** Acute change in ICP over time (hours), indexed to the time of central line insertion (time 0). **b** Subacute change in ICP over time (hours), related to the first ICP measurement (time of registration, time 0) up to 10 days after insertion. **c** Subacute change in ICP as in b, but restricted to a high-risk subgroup (WFNS grades 4–5). **d** Subacute change in ICP as in b, but restricted to patients with at least two documented measurements of ICP > 20 prior to CVC insertion. Shaded area represents the 95% confidence interval

Acute changes in ICP were compared by indexing the ICP at catheter insertion time and comparing hourly ICP measurements for up to 3 h before insertion through 24 h after insertion. Catheter insertion location did not modify ICP measurements in the acute setting (Fig. 2A).

To evaluate subacute ICP complications after CVC placement, we analyzed ICP measurements indexed from EVD placement through the following 10 days or when the EVD was removed, whichever came first. There was no significant difference in ICP between groups over this interval (Fig. 2B). To assess whether jugular catheterization may precipitate elevated ICP in patients at the highest risk for ICP complications, ICP curves were analyzed in a subset of patients with high-grade presentations at highest risk for ICP complications (WFNS 4–5, 104 IJ vs. 318 non-IJ patients, Fig. 2C), and a subset of patients who had two or more ICP values above 20 before CVC insertion (22 IJ vs. 54 non-IJ patients, Fig. 2D). There was no association of ICP with CVC location in these two subsets.

## Discussion

Classic teaching in neurocritical care has been to avoid jugular CVC access due to a risk of precipitating ICP complications in patients at a high risk of intracranial hypertension. Using a large, retrospective cohort analysis of both ICP analysis and catheter-associated complications, we found that jugular venous access is not associated with elevated ICP in this clinical context and may be associated with decreased catheter-associated complications.

Our study is unique in providing ICP trend analysis in 1,035 patients and directly assessing whether CVC location modified ICP in patients at risk for diffuse cerebral edema. To our knowledge, this is the only quantitative study assessing the role of CVC placement on ICP measurements over a large cohort of neurocritically ill patients. This granular ICP analysis is consistent with anatomical insights of cerebral venous outflow.

First, venous outflow obstruction leading to increased cerebral blood volume and ICP is unlikely given basic catheter size considerations. A typical 7 French triple lumen CVC has an outer diameter of 2.33 mm, whereas the diameter of the IJ vein is typically greater than 12 mm [21, 22]. Venous outflow obstruction is highly unlikely with vessel lumen occupancy well below 50%, even when accounting for large bore dialysis catheters with an outer diameter of 4.4 mm. Although superimposed thrombosis could certainly impede or obstruct venous outflow, these are rare events that occur in up to 1% of patients (without further qualification of the severity and thus obstruction) [15]. We observed a 0.5% incidence of caDVT with IJ access, but this is likely confounded by the lack of systemic surveillance. Although a notable limitation, this level of thrombosis would likely be detected by ICP analysis if severe. Our analysis could not detect, however, an immediate and transient increase in ICP because our data were sourced from the hourly routine ICP charting. Nevertheless, the lack of immediate ICP elevations with jugular catheterization has been previously reported [12, 13].

Second, cerebral venous outflow is more complicated than jugular outflow. When in a supine position, the majority of venous outflow is through the jugular venous system, whereas the jugular veins are typically collapsed when upright. In the upright position, extra-jugular venous outflow tracts are the dominant outflow route, draining blood through multiple anastomotic channels to the caval venous system [23–25]. These collateral outflow channels are of particular interest in patients with intracranial hypertension, as these patients are typically positioned with an elevated head-of-bed and may be least dependent on jugular venous outflow.

Our data further suggest a preference for ultrasound-guided IJ CVC placement in this cohort given the low rate of catheter complications. The lower rate of complications was largely driven by pneumothorax with SC CVCs and caDVT with PICCs and femoral CVCs. SC venous cannulation was common in our cohort but associated with increased risk of traumatic pneumothorax. Although the rates reported herein are below acceptable risk, the use of ultrasound-guided subclavian venous cannulation (which was not used in our cohort) may further reduce this risk [26, 27]. It is certainly possible that use of ultrasound guidance, rather than venous access site selection, drives the lower rates of catheter complications when comparing IJ and SC access sites (Table 2).

**Table 2:** Complication rates per central line insertion site

Access Site	Total	Total Catheter Days	Average Length [Days, IQR]	PTX	caDVT	CLABSI	Any Event	p value	Any event per day	p value
Internal Jugular	365	3796	10 (6–14)	1 (0.3)	2 (0.5)	3 (0.8)	6 (1.6)	** < 0.001**	0.0016	** < 0.001**
Non-IJ	1212		10 (6–15)	27 (2.2)	23 (1.7)	10 (0.8)	56 (4.8)		0.0462	
*Subclavian*	*887*	10,303	12 (7–15)	*23 (2.6)*	*8 (0.9)*	*7 (0.8)*	*38 (4.2)*		0.0428	
*Femoral*	*72*	248	3 (1–4)	*0*	*6 (8.3)*	*0*	*6 (8.3)*		0.0833	
*PICC*	*253*	2996	12 (5–15)	*0*	*9 (3.6)*	*3 (1.2)*	*12 (4.7)*		0.004	

*p* value comparing internal jugular (IJ) placement versus all other sites (non-IJ). Below is the breakdown of non-IJ sites

*caDVT* catheter-associated deep venous thrombosis; *caDVT* catheter-associated deep venous thrombosis; *CLABSI* central-line associated blood stream infection; *PTX* pneumothorax

PICC placement has recently grown in popularity as a safe alternative, although controversy persists [28, 29]. Our data deliver a clear warning regarding the rate of caDVT associated with PICC lines in neurocritically ill patients. Although upper extremity DVT has often been thought to be less dangerous than lower extremity DVT, registry data demonstrate comparable risk of pulmonary embolism and mortality, even with catheter-associated upper extremity DVT [30, 31]. Further, treatment requires anticoagulation, which increases the risk for hemorrhagic complications. In our cohort, this includes the risk of EVD-related tract hemorrhages, whereas in other neurocritically ill patients, this includes the risk of hemorrhagic conversion of infarcted tissue. Our data differ from a recent, randomized controlled trial comparing catheter complications of PICC and CVC in neurocritically ill patients, in which similar outcomes but a nonsignificant trend toward increased complications with PICC placement was reported [32]. The relative difference between our experiences is likely due to study size and power. Together, these data highlight the unintended risks associated with PICC placement in neurocritically ill patients, both in terms of venous thromboembolism and the prospect of anticoagulation dilemmas.

Although our study benefits from its size and detailed patient-level data, it has several limitations. First, it is retrospective in nature and not randomized. The selection of CVC location was likely confounded by indication. For example, femoral access in our practice is typically used in extremis or as a last resort (eg, if a patient cannot tolerate lying flat due to elevated ICP and the jugular vein is collapsed when seated upright), likely instilling a selection bias against femoral placement. We found no such confound for selection of SC versus IJ placement, although habitus and operator preference likely played a role. Second, surveillance for venous thromboses was clinically driven and not part of systematic surveillance. Therefore, the true incidence of DVT is likely higher than reported. Further, sampling bias likely confounds the relative rates of caDVT in femoral CVC given the increased frequency of lower extremity venous studies. Nevertheless, this would not explain the increased rates of caDVT in PICCs. Third, although aSAH typically precipitates diffuse cerebral edema, a small subset of patients will have associated parenchymal hematomas. These could cause lateralizing foci of compression, which could theoretically interact with the laterality of the IJ catheter. Fourth, the relative low incidence of ICP crisis may limit the sensitivity of this analysis. Although subgroup analysis comparing patients at the highest risk for ICP crisis (either by high-grade presentation or sustained ICP elevation) did not detect a difference at a population level, it remains possible that specific patients with unique anatomical considerations may be at risk for jugular access complications and intracranial hypertension. Finally, another potential contributor to the lack of increase in ICP with IJ CVC placement could be related to our unit’s standard of care and heterogenous treatment effects. Maintaining an open EVD and using osmotherapy likely reduce the risk for elevated ICP events and decrease sensitivity to detect a difference if present. Further, maintaining an open EVD has the potential to increase the effective compliance of a system that would otherwise be less compliant, and therefore decrease the sensitivity for ICP changes associated with jugular catheterization and/or changes in cerebral blood volume. This may limit the generalizability to other noncompliant, higher ICP systems, depending on the clinical context. To test if these care practices had a protective effect against ICP elevations, future studies can perform a similar analysis in a setting in which the default is to maintain the EVD clamped or to use a cohort of patients with parenchymal ICP monitors and not EVDs.

## Conclusions

Contrary to classic teaching, we find that IJ CVC placement was not associated with increased ICP in the clinical context of this large, single-center retrospective cohort. Further, ultrasound-guided IJ access was less likely to be associated with an access-site complication when compared with non-IJ access sites. PICC placement was associated with high rates of DVT. Together, these data support the safety and at least noninferiority of ultrasound-guided IJ venous catheterization in patients with aSAH.

## Supplementary Information

Below is the link to the electronic supplementary material.

Supplementary file1 (DOCX 30 KB)
