# Supplementary material for: Jugular Venous Catheterization is Not Associated with Increased Complications in Patients with Aneurysmal Subarachnoid Hemorrhage
Source: Neurocrit Care. 2024 Nov 26;42(3):929–36. doi: 10.1007/s12028-024-02173-1 (PMC12137458; doi:10.1007/s12028-024-02173-1)
Supplement: Supplementary file 1 — Supplementary file1 (DOCX 30 KB) [file 12028_2024_2173_MOESM1_ESM.docx]

**Supplementary methods**

We assume that there are $n$ subjects, and the $i$-th subject takes $m_{i}$ measurements within the time window of interest. This was denoted by the observed data as $\{\left( x_{i},t_{i,j}, Y_{i,j} \right):i=1,\ldots,n, j=1,\ldots,m_{i}\}$, where $x_{i}$ is a binary variable (0 or 1) denoting whether the $i$-th subject belongs to the IJ or non-IJ cohort, $t_{i,j}$ is the time of the $j$-th ICP measurement of the $i$-th subject, and $Y_{i,j}$ is the observed value of the ICP measurement at time $t_{i,j}$. We then fit the following mixed effect model:

$$Y_{i,j}=\beta_{0}+\beta_{1}t_{i,j}+\beta_{2}x_{i}+\beta_{3}x_{i}t_{i,j}+b_{i}+\epsilon_{i,j},$$

$$b_{i}\sim N\left( 0,\sigma_{b}^{2} \right), \epsilon_{i,j}\sim N\left( 0,\sigma^{2} \right).$$

Here $\beta_{0}$ denotes the average base ICP value at time 0 within the IJ cohort, $\beta_{1}$ denotes the average slope of ICP within the IJ cohort, $\beta_{2}$ and $\beta_{3}$ denote the difference in the average base ICP and slope between the IJ and non-IJ cohort, $b_{i}$ is the random effects associated with the $i$-th subject, and $\epsilon_{i,t}$’s are the i.i.d. random noise. To compare the ICP curves for two cohorts, we report the p-values of $\beta_{2}$and $\beta_{3}$ for testing the null-hypotheses that $\beta_{2}=0$ and $\beta_{3}=0$, which indicate the significance of the difference between the two cohorts.

**Supplementary results**

**Supplementary Table S1. Central venous catheter placement by attempt.**

|  | **Catheter Placement** | | | | | |
| --- | --- | --- | --- | --- | --- | --- |
| **Access Site** | **1st** | **2nd** | **3rd** | **4th** | **5th** | **Total** |
| Internal Jugular | 304 | 50 | 9 | 2 | 1 | 366 |
| Subclavian | 808 | 66 | 11 | 2 | 0 | 887 |
| Femoral | 40 | 23 | 7 | 2 | 0 | 72 |
| PICC | 76 | 146 | 25 | 5 | 2 | 254 |

**Supplementary Table S2. Duration of placed central venous catheters by attempt, in days.**

|  | **Catheter Placement** | | | | | | **Days per Patient (IQR)** |
| --- | --- | --- | --- | --- | --- | --- | --- |
| **Access Site** | **1st** | **2nd** | **3rd** | **4th** | **5th** | **Total** |  |
| Internal Jugular | 3,238 | 445 | 80 | 24 | 9 | 3,796 | 10 (6-14) |
| Subclavian | 9,460 | 720 | 105 | 18 | 0 | 10,303 | 12 (7-15) |
| Femoral | 87 | 114 | 40 | 7 | 0 | 248 | 3 (1-4) |
| PICC | 774 | 1,684 | 417 | 73 | 48 | 2,996 | 12 (5-15) |

**Supplementary Table S3**:  **Patient demographics and clinical characteristics by internal jugular (IJ) versus all other sites (non-IJ)**.

|  | IJ | Non-IJ | P-value |
| --- | --- | --- | --- |
| Demographics | |  |  |
| Patients | 303 | 920 |  |
| Age (mean & IQR) | 53.4 (44.0-62.2) | 54.5 (46.0-63.0) | 0.14 |
| Female | 67.3% [62.1-72.6] | 70.5% [67.6-73.5] | 0.313 |
| Weight (mean & IQR) | 101.3 (69.9-115.3) | 95.1 (65.9-109.3) | |
| BMI (mean & IQR) | 32.1 (23.8-37.1) | 31.8 (23.1-36.9) | |
| Race |  |  | **0.006** |
| White | 35.0% [29.6-40.4] | 41.2% [38.0-44.4] | |
| African American | 44.6% [39.0-50.2] | 33.8% [30.8-36.9] | |
| Asian | 2.0% [0.4-3.6] | 3.8% [2.6-5.0] | |
| Other | 18.5% [14.1-22.9] | 21.2% [18.6-23.8] | |
| Hypertension | 58.1% [52.5-63.6] | 56.9% [53.7-60.1] | 0.738 |
| Diabetes Mellitus | 16.2% [12.0-20.3] | 11.9% [9.8-13.9] | 0.060 |
| Smoker | 24.1% [19.3-28.9] | 30.2% [27.3-33.2] | **0.041** |
| Coronary artery disease | 8.3% [5.2-11.4] | 6.3% [4.7-7.9] | 0.238 |
| Hypercholesterolemia/dyslipidemia | 15.2% [11.1-19.2] | 15.0% [12.7-17.3] | 0.926 |
|  |  |  |  |
| Presentation |  |  |  |
| SAH |  |  | **0.006** |
| Aneurysmal SAH | 79.5% [75.0-84.1] | 86.4% [84.2-88.6] | |
| Angiogram negative SAH | 20.5% [15.9-25.0] | 13.6% [11.4-15.8] | |
| WFNS scale (median & IQR) | 2 (1-4) | 2 (1-4) | 0.822 |
| Modified Fisher scale (median & IQR) | 4 (3-4) | 4 (3-4) | 0.220 |
| Surgical treatment (for aneurysmal SAH) | | | 0.414 |
| Clip | 24.8% [19.9-29.6] | 23.5% [20.7-26.2] | |
| Coil | 46.5% [40.9-52.2] | 54.7% [51.5-57.9] | |
| None | 8.3% [5.2-11.4] | 8.5% [6.7-10.3] | |

**Supplementary Table S4**: **Complication rates per central line insertion site**.

| **Access Site** | **Total (n)** | **Total Catheter Days** | **Average Length [Days, IQR]** | **PTX** | **caDVT** | **CLABSI** | **Any Event** | **p value** | **Any event per day** | **p value** |  |
| --- | --- | --- | --- | --- | --- | --- | --- | --- | --- | --- | --- |
| **Internal Jugular** | 365 | 3,796 | 10 (6-14) | 1 (0.3) | 2 (0.5) | 3 (0.8) | 6 (1.6) | **<0.001** | 0.0016 | **<0.001** |  |
| **Non-IJ** | 1,212 |  | 10 (6-15) | 27 (2.2) | 23 (1.7) | 10 (0.8) | 56 (4.8) | | *0.0462* |  |  |
| ***Subclavian*** | *887* | 10,303 | 12 (7-15) | *23 (2.6)* | *8 (0.9)* | *7 (0.8)* | *38 (4.2)* | | *0.0428* | | |
| ***Femoral*** | *72* | 248 | 3 (1-4) | *0* | *6 (8.3)* | *0* | *6 (8.3)* | | *0.0833* | | |
| ***PICC*** | *253* | 2,996 | 12 (5-15) | *0* | *9 (3.6)* | *3 (1.2)* | *12 (4.7)* | | *0.004* | | |

p value comparing internal jugular (IJ) placement versus all other sites (non-IJ). Below is the breakdown of non-IJ sites. caDVT, catheter-associated deep venous thrombosis; caDVT, catheter-associated deep venous thrombosis; CLABSI, central-line associated blood stream infection; PTX, pneumothorax

**Supplementary Table S5**:  **Frequency of radiographic surveillance for thrombotic complications by site and frequency of positive studies per access site.**

| **Venous Thrombosis Study** | **Studies** | **Catheter Days for Studied Sites** | **Studies/catheter day** |
| --- | --- | --- | --- |
| Lower extremity venous US | 829 | 248 | 3.34 |
| Upper extremity venous US | 234 | 17,095 | 0.01 |
| Neck Angiography (US, CT, MR) | 198 | 3,796 | 0.05 |
|  |  |  |  |
| **Central Venous Catheter Site** | **Studies Assessed** | **caDVT** | **Studies Positive (%)** |
| Internal Jugular | 432 | 2 | 0.46 |
| Subclavian | 234 | 8 | 3.42 |
| Peripherally Inserted Central Catheter | 234 | 9 | 3.85 |
| Femoral | 829 | 6 | 0.72 |

**Supplementary Table S6**:  **Binary logistic regression comparing access sites to incidence of catheter associated deep venous thrombosis, indexed to the internal jugular rates.**

| **Variable** | **aOR** | **p value** |
| --- | --- | --- |
| PICC | 7.22 (1.19 - 43.90) | 0.032 |
| Subclavian | 2.82 (0.64 - 12.39) | 0.17 |
| Femoral | 14.81 (2.81 - 77.94) | 0.001 |
